# Diagnosing filamentous fungal infections in immunocompromised patients applying computed tomography-guided percutaneous lung biopsies: a 12-year experience

**DOI:** 10.1007/s15010-017-1072-6

**Published:** 2017-09-27

**Authors:** Cornelia Lass-Flörl, Maria Aigner, David Nachbaur, Stephan Eschertzhuber, Brigitte Bucher, Christian Geltner, Romuald Bellmann, Michaela Lackner, Dorothea Orth-Höller, Reinhard Würzner, Günter Weiss, Bernhard Glodny

**Affiliations:** 1 Division of Hygiene and Medical Microbiology, Medical University of Innsbruck, Schöpfstraße 41/III, 6020 Innsbruck, Tirol Austria; 2 Department of Radiology, University Hospital Innsbruck, Innsbruck, Austria; 3 Division of Haematology and Oncology, Medical University of Innsbruck, Innsbruck, Austria; 4 Transplant Intensive Care Unit, Department of Anesthesia and Critical Care, Centre of Operative Medicine, Medical University of Innsbruck, Innsbruck, Austria; 5 Department of Pulmonology, Hospital Natters, Natters, Austria; 6 Division of Intensive Care and Emergency Medicine, Department of Internal Medicine I, Medical University of Innsbruck, Innsbruck, Austria; 7 Department of Internal Medicine II, Medical University of Innsbruck, Innsbruck, Austria

**Keywords:** *Aspergillus*, Mucormycoses, Fungal infection, Invasive fungal disease, Computed tomography-guided lung biopsy

## Abstract

**Background:**

Invasive fungal diseases (IFD) are an important cause of morbidity and mortality in immunocompromised patients, and early diagnosis and management are a challenge. We evaluated the clinical utility of computed tomography (CT)-guided percutaneous lung biopsies in diagnosing IFD.

**Methods:**

Between 2003 and 2014, we analyzed 2671 CT-guided lung biopsies, from which 157 were IFD associated; we aimed to determine microbiological-based diagnostic accuracy of calcofluor white staining (CFWS), culture, *Aspergillus* antigen detection (GM), broad-range fungal PCR, and *Aspergillus* PCR per sample.

**Results:**

127 (81%) specimens were microscopically positive for any fungal elements, 30 (19%) negative. *Aspergillus* and non-*Aspergillus* like hyphae were obtained in 85 (67%) and 42 (33%) specimens, respectively. CFWS positivity was defined as proof of infection. Sensitivity, specificity, and positive (PPV) and negative predictive (NPV) values for CT scan were 100, 44, 80, and 100%, for *Aspergillus* PCR 89, 58, 88, and 58%, for broad-range fungal PCR 90, 83, 95, and 90%, and for GM 94, 83, 95, and 90%. The most common CT features were patchy opacifications with central necrosis (78%) or cavern defects (50%), less common were air bronchograms (39%) or ground glass halos (39%), and all other features were rare. The overall pneumothorax rate subsequent to biopsy was 19%, but in only 2% of all cases the placement of a chest tube was indicated. One case of fatal air embolism occurred.

**Conclusions:**

CT-guided lung biopsies have high diagnostic accuracy in terms of microscopic examination, and complication rates are low. Molecular-based and antigen tests applied on fungal hyphae-positive specimens showed comparable results.

## Introduction

Invasive fungal diseases (IFD) are a major cause of morbidity and mortality in immunocompromised patients [1]. Case fatality rates range from 30 to 80% in neutropenic patients and result at least partly from difficulties in obtaining a reliable diagnosis at an early stage of disease. The diagnostic workup may consist of imaging techniques, culture, serology, and molecular-based methods. Culture-based methods are often delayed and may have low sensitivity [2], because the materials used are not picked up from the proper site of ongoing, progressive infection yielding necrotic tissue. In most instances, an antimycotic treatment had already been started, so that the viability of the pathogens is hampered. Galactomannan detection in body fluids is more sensitive than culture for diagnosis of invasive aspergillosis (IA), but sensitivity is variable (17–100%) [2]. Only moderate data are available for *Aspergillus* PCR [3] and β-d-glucan (Fungitell, Associates of Cape Cod) [4] in diagnosing IFD, from blood and broncho-alveolar lavages (BAL). Imaging has a crucial role in the diagnosis and management of patients with suspected IFD who are immunocompromised [2]. The preferred method of choice is a computed tomography (CT) scan, as chest radiography might show normal or non-specific findings in neutropenic patients with IFD [5]. Although the halo sign and air crescent sign are characteristic, they are not diagnostic of IA. Infections due to other angioinvasive fungi, such as Mucorales, *Fusarium* spp. as well as *Nocardia* spp. may cause radiological features described for IA [6, 7]. However, the identification of the causative organism is highly warranted for a targeted treatment, especially if patients fail to respond to standard anti-mold therapy. Mucorales are resistant to the newer antifungal drugs such as voriconazole and echinocandins [8] and therefore the choice of first-line treatment is (liposomal) amphotericin B; *A. terreus*, by contrast, is amphotericin B resistant; both fungal pathogens are frequently involved in pulmonary infections in our patient cohort [9–11]. Hence, our center needs a powerful diagnostic strategy with the performance of CT-guided lung biopsies displaying a standard of care. Here, we aimed to investigate the value of CT-guided lung biopsies in diagnosing IFDs in immunocompromised patients and best practice specimen handling, applying Calcofluor White staining (CFWS), culture, *Aspergillus* antigen detection (GM), broad-range fungal PCR, and *Aspergillus* PCR in lung specimens obtained during 2003–2014.

## Materials and methods

### Patients

We attended to 2671 patients undergoing lung biopsy for any diagnostic reason, including cancer, infectious diseases, and others between 2003 and 2014. Of them, 179 patients were evaluated with fever and a CT scan compatible with filamentous fungal lung infections according to EORTC/MSG criteria [12]. Of these patients, 168 eligible patients underwent CT-guided lung biopsies because they either did not improve during standard antifungal therapy or the proof of diagnosis was important for further medical procedures. From this cohort, 11 samples were excluded for various reasons such as lack of triple diagnostic tests performed (*n* = 6), PCR inhibition (*n* = 2), or lack of detailed clinical data (*n* = 3). Thus, data were evaluated of 157 patients, with the majority having underlying hematological malignancies (*n* = 85), solid organ transplantations (*n* = 40), solid cancer (*n* = 19) or otherwise severe immunocompromised conditions (*n* = 13). Lung biopsy specimens were investigated for the presence of fungal pathogens.

Patients received standard of care treatment, including antibiotics and antifungals, and those with hematological malignancies and or allogeneic/autologous stem cell transplantations and or lung transplantation received standard prophylaxis with fluconazole, and/or posaconazole, and/or micafungin if indicated [13]. CT scans were performed as part of the diagnostic workup and were reviewed by a senior radiologist for evidence of fungal infection, which included the presence and distribution of nodule(s) and/or consolidations with or without ground-glass opacities, a halo sign, and an air crescent sign and cavitation. Various CT scanners of different vendors and protocols with various slice thickness with or without intravenous contrast enhancement were used over the time. In all patients, the whole chest was scanned continuously utilizing a multidetector spiral CT technique, including a high-resolution mode with at least 1.0 mm or even sub-millimeter slice thickness.

Patients who were hemodynamically unstable or in whom platelet counts could not be maintained over 60 × 10^9^/l were excluded. Thrombocytopenic patients received platelet transfusions before the procedure, at the discretion of the clinical team, and a prothrombin time of at least 50% (normal range 70–130%) was requested. Patients suspected to suffer from IFDs were on empirical antifungal therapy at the time of taking lung biopsies.

### CT-guided percutaneous lung biopsies

Lung biopsies were performed by different radiologists, exclusively using CT. Two CT devices, equipped with the Smart-Step technique, were deployed for this purpose (Lightspeed 16 or VCT 64, GE Healthcare) [14]. Decisions for patient management were made on a case-by-case basis by the radiologists, based on size and location of the pulmonary lesion. After the intervention, patients were routinely monitored and initially chest radiographs and after 2007 low-dose CTs of the entire chest thorax [15] were obtained during follow-up to exclude complications, particularly pneumothorax.

CT-guided percutaneous biopsies were performed with an automated biopsy gun with a detachable coaxial cutting needle system (Bard^®^) as described by Lucidarme et al. [16]. In our study, an outer coaxial needle with 18-gauge diameter and an inner biopsy needle with 17-gauge diameter were chosen with a local anesthesia with 1% lidocaine, either without or, in most cases, with general anesthesia. On average, 8.5 ± 3.3 samples were taken, with a length of either 1.5 or 2.2 cm. Clinical specimens were collected at the Division of Hygiene and Medical Microbiology, Medical University of Innsbruck, and samples were aseptically divided into three fractions for microscopy, culture, PCR, and GM testing.

### Fungal microbiology

**Fig. 1:**
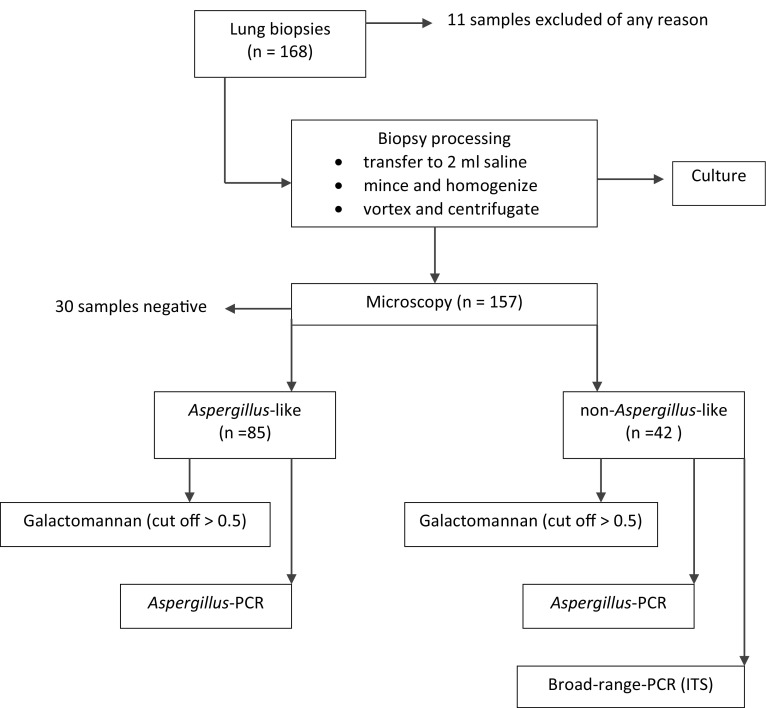
Flow diagram of the sequence of diagnostic test applied

Biopsy specimens, transferred to 2 ml normal saline were minced and homogenized aseptically. Afterward, samples were vortexed, kept at room temperature for 30 min, and centrifuged. All specimens, consisting of supernatants and homogenized tissues, were investigated for fungi by application of the Fungi-Fluor™ (Calcofluor White staining solution, Polysciences, USA; CFWS staining). Samples which showed *Aspergillus*-like hyphae were tested for GM and *Aspergillus* PCR [17, 18] positivity. GM (BioRad, Austria) was performed according to the instructions of the company and a 0.5 cutoff was used to define the positivity for GM. Samples which showed non-*Aspergillus*-like hyphae by CFWS and which remained negative in GM and *Aspergillus* PCR were evaluated by a broad-range PCR using internally transcribed spacer region (ITS) [19]. In parallel, all samples were cultured in Sabouraud Dextrose Broth at 37 °C for 14 days. For controls, 24 CT-guided lung biopsies not associated with any IFD were evaluated applying all microbiological assays. Figure 1 shows a flow diagram of the sequence of diagnostic tests applied.

### Criteria for fungal diagnosis

Proven pulmonary IA was defined as positive tissue biopsy showing typical septate, acute branching hyphae (CFWS) with positive GM and *Aspergillus* PCR tests and with or without positive cultures for *Aspergillus*. Proven pulmonary IFD (non-*Aspergillus* fungal infection) was defined as positive tissue biopsy showing unseptate or sparsely septate, broad, and irregular hyphae with positive panfungal PCR tests and with or without positive culture for any suitable fungus. Samples with negative results were reported as sterile; samples showing technical difficulties were excluded from the analysis (*n* = 11).

### Statistical analysis

The primary purpose of the study was to evaluate the feasibility of lung biopsies in diagnosing fungal infections in immunocompromised patients. The secondary purposes were to determine the outcome in terms of diagnostic yield and to determine the complications related to the procedure. The Society of Interventional Radiology clinical practice guidelines of 2003 were used to classify minor and major complications [20]. Statistical analysis included a *t* test for assessing the difference in continuous values between the two groups (i.e. patients with *Aspergillus* infections and patients with non-*Aspergillus* filamentous fungal infections); a Chi square test was used to assess the difference in categorical values between the two groups. A *p* value < 0.05 was considered to be statistically significant.

## Results

**Fig. 2:**
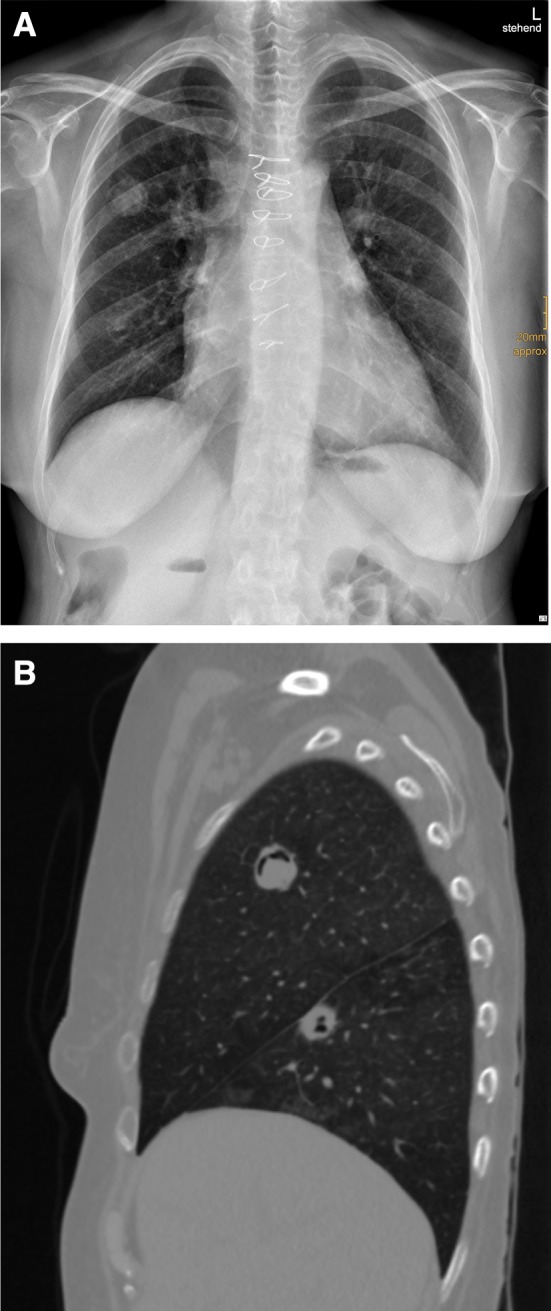
X-ray and computer tomographic images of a 52-year-old immunocompromised patient after heart transplantation suffering from pulmonary aspergillosis (*Aspergillus terreus).* **a** Several lesions on both lungs on X-ray with two of the lesions **b** showing an air crescent sign (sagittal reconstruction). The lesion located in the lower lobe developed a ground-glass halo later on, and no pleural effusion is seen

Using CFWS, 127 (81%) samples were positive for any fungal elements, 30 (19%) remained negative. *Aspergillus-* and non-*Aspergillus*-like hyphae were obtained in 85 (67%) and 42 (33%) specimens, respectively (Table 1 and Fig. 2). Fungal species identification done by micromorphology or molecular-based methods yielded *Aspergillus* spp., Mucoraceae and others (Table 2); cultures were positive in only 62 samples investigated. Control specimens remained culture, *Aspergillus* PCR, broad-range PCR, and GM antigen test negative in all cases. CFWS positivity, which reflects proof of an infection according to EORTC criteria (golden standard), was defined as the standard of comparison; sensitivity, specificity, and positive (PPV) and negative predictive (NPV) values for CT scan, *Aspergillus* PCR, broad-range PCR, and GM from lung biopsy specimens are outlined in Table 3. Antifungal therapy at time of biopsy consisted of voriconazole and/or echinocandins in 55% of patients suffering from non-*Aspergillus* infections, hence displaying lack of activity against fungal pathogens identified. Over the years, a shift from *Aspergillus* to non-*Aspergillus* infections took place and, based on antifungal treatment strategies applied, it is obvious that anti-mold prophylaxis successfully prevents IA, as only five cases were detected during 2011 and 2014. In comparison, 80 IA cases were detected between 2003 and 2010, respectively, see Table 4.

**Table 1:** Characteristics of 127 patients with invasive pulmonary fungal infections microscopically diagnosed by immunofluorescence staining of lung biopsies in 2000–2015

Characteristics	No. of patients (%)		*P* value^a^
	*Aspergillus-*like hyphae	Non-*Aspergillus-*like hyphae	
Patients	85 (67)	42 (33)	
Female gender	18 (50)	7 (54)	0.86
Mean of age in years ± SD	63 ± 4	59 ± 7	0.09
Underlying diseases^b^			0.11
Hematological malignancies	41 (32)	30 (24)	
Solid organ transplantations	36 (28)	2 (2)	
Solid tumors	5 (4)	7 (6)	
Others	3 (2)	3 (2)	
Antifungal therapy at the time of biopsy intervention			0.27
Voriconazole	43 (34)	6 (46)	
Echinocandins^c^	21 (17)	11 (9)	
Lipid-amphotericin B	18 (14)	5 (4)	
Posaconazole	3 (2)	12 (9)	

^a^ Fisher’s exact test, Student’s test, Chi squared test

^b^ Including patients suffering from acute myeloid leukemia, acute lymphoid leukemia, myelodysplastic syndrome, lymphoma, and lung transplantations

^c^ Including micafungin and caspofungin

**Table 2:** Genus and species identification from 127 fungal-positive lung biopsies obtained by CT-guided procedures

Species	No. of patients (%)
*Aspergillus fumigatus* complex	35 (27.5)
*Aspergillus terreus* complex	44 (34.6)
*Aspergillus flavus* complex	2 (1.5)
other *Aspergillus* species	4 (3.1)
*Mucor* sp.	11 (8.6)
*Rhizomucor* sp.	9 (7.0)
*Rhizopus* sp.	5 (3.9)
*Lichtheimia corymbifera*	6 (4.7)
*Cunninghamella* sp.	4 (3.1)
*Scedosporium* sp.	2 (1.5)
*Penicillium* sp.	3 (2.3)
*Trichosporon* sp.	2 (1.5)

Genus and species identification was obtained by culture and micromorphology typing or by applying PCR targeting the ITS techniques

**Table 3:** Performance of various diagnostic assays in relation to fungal-positive lung biopsies obtained from invasive CT-guided interventions

Test assays	% Sensitivity	% Specificity	% PPV	% NPV
CT scan	100	44	80	100
*Aspergillus* PCR^a^	89	58	88	58
Broad-range fungal PCR^b^	90	83	95	90
GM^c^	94	83	95	90

*PPV* positive predictive value, *NPV* negative predictive value

CT-guided lung samples resulted from 127 patients and 24 control (negative) patients

^a^ Over the last 12 years, we used various *Aspergillus-*specific PCR assays targeting the 18S ribosomal RNA

^b^ We applied a broad-range PCR using the internal transcribed spacer region

^c^ GM testing defined a cutoff value of 0.5

**Table 4:** Fungal pathogens identified, treatment modalities, and diagnostic tests applied over the last 12 years in immunosuppressed patients

Time period	2003–2006	2007–2010	2011–2014
Number of patients with proven lung infections	55^a^	43	29
Fungal pathogens identified			
*Aspergillus* species	42	38	5
*A. terreus* complex	24	19	1
*A. fumigatus* complex	18	16	1
*A. flavus* complex	0	1	1
Other species	0	2	2
Mucorales	5	12	18
Others	0	1	6
Main antifungal treatment strategies applied	Empirical and pre-emptive treatment^b^	Empirical and pre-emptive treatment^c^	Anti-mold prophylaxis^d^
Laboratory blood screening tests performed	*Aspergillus*-specific PCR	GM *Aspergillus*-specific PCR Panfungal PCR	None

*GM* galactomannan testing, *PCR* polymerase chain reaction

^a^ Data obtained from few patients have been reported earlier in 2007 [9]

^b^ Mainly, empirical treatment was undertaken applying amphotericin B or caspofungin

^c^ Due to the extensive performance of polymerase chain reaction assays (various protocols) and GM blood screenings, pre-emptive treatment strategies with voriconazole and/or caspofungin were applied

^d^ Antifungal mold prophylaxis with micafungin and posaconazole was administered to patients at risk

**Fig. 3:**
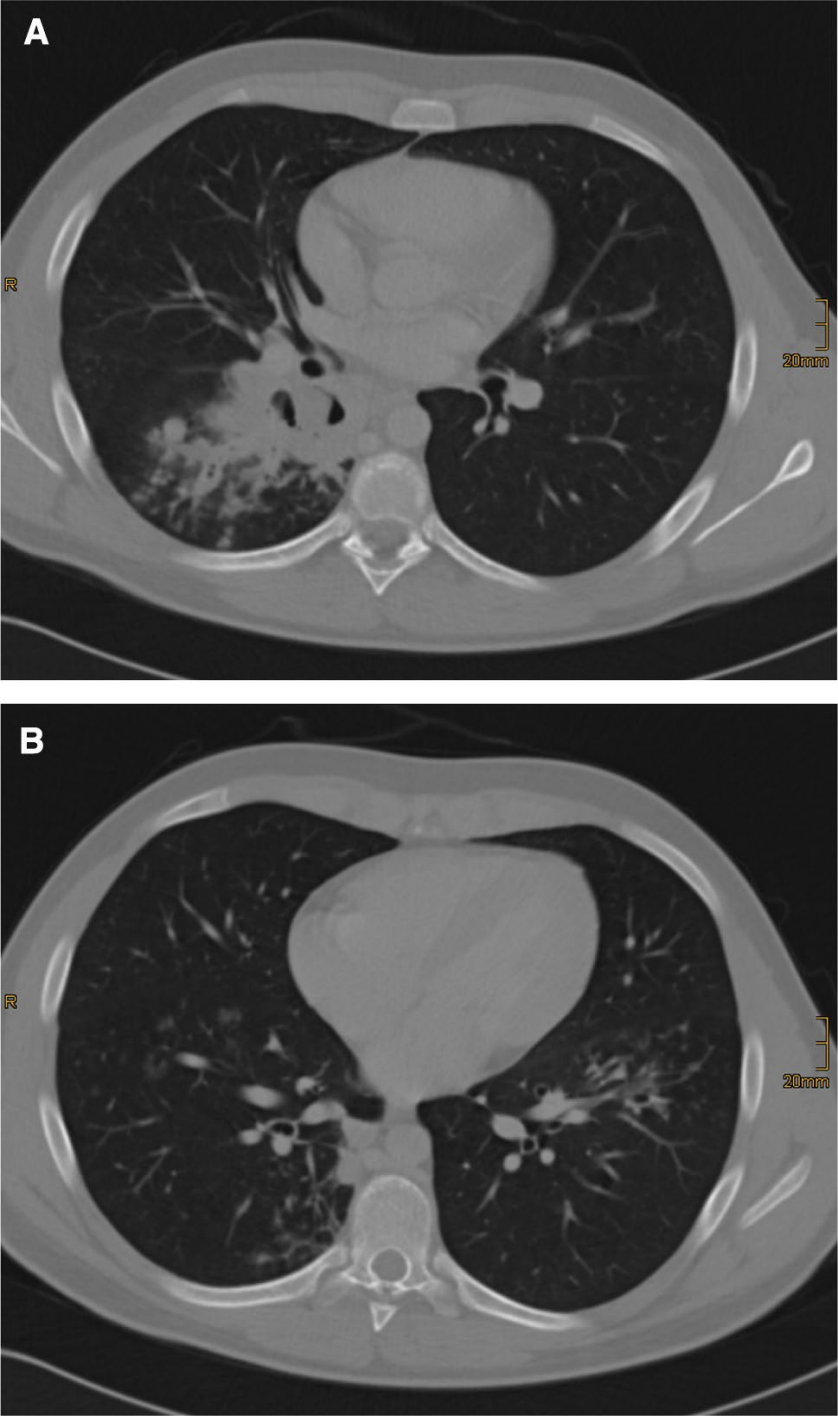
Chest computer tomographic scan of a 21-year-old patient suffering from acute lymphatic leukemia and mucormycosis. **a**, A cavern is seen in the right lower lobe of the lung adjacent to the hilum, accompanied by more peripheral small nodules and tree in bud phenomenon, but also in the other lobes of the lung as well, representing a mucormycosis. **b**, The right lower lobe was removed surgically and the patient recovered fully

**Fig. 4:**
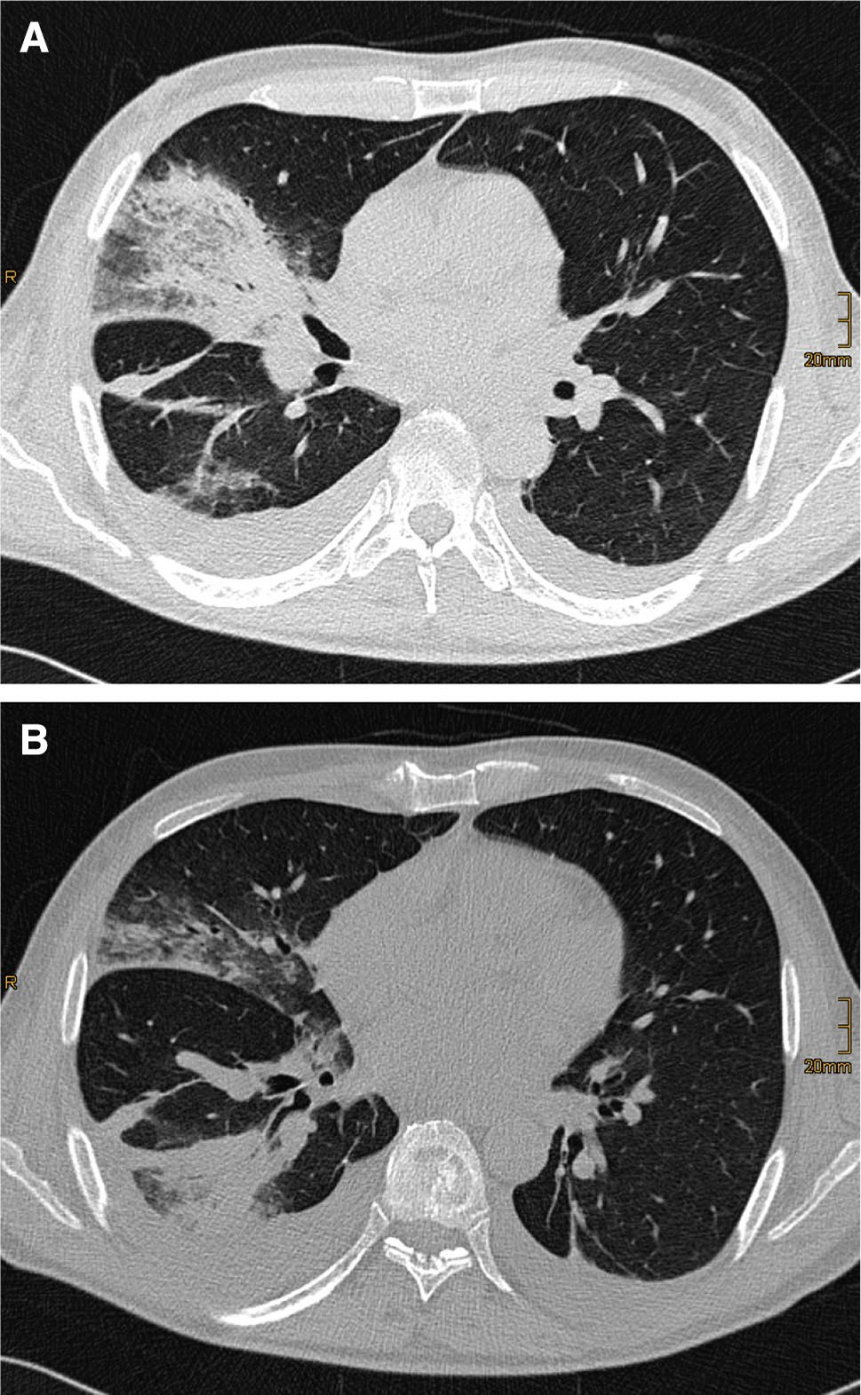
Chest computer tomographic lung scan of a 42-year-old patient during aplasia following therapy for acute lymphatic leukemia and suffering from proven mucormycosis. **a**, Air space opacification with positive bronchopneumogram in the right upper and **b**, lower lobe accompanied by parapneumonic effusions on both lung sides

Patients with mycosis were younger than patients without mycosis (50.3 ± 19.3 versus 62 ± 14 years, *p* < 0.0001). Mycotic lesions were located in the upper lobes of the lungs in 59% of the cases, 33% in the lower lobes, and 8% in the middle lobe or lingula. The most common CT features of the lesions at the time of biopsy were patchy opacifications with central necrosis (78%) or cavern defects (50%), and less common were air bronchograms (39%), or ground-glass opacity halos (39%), see Figs. 3 and 4. Extensive alveolar space opacities were present in 41% of cases, in combination with patchy opacities in 15%, and without patchy opacities in 26%. Tree in bud phenomenon was rare (13%), as well as other features. The typical air crescent sign was found in only 32% of patients; this low percentage of the air crescent sign is probably because the decision to perform a lung biopsy is made only after a certain delay, so that the early sign fails to be detected.

Mycotic lesions showed significantly lower densities than non-mycotic lesions; they were larger and located more peripherally in the lung (*p* < 0.0001 each). The minimal distance between the lesion and the parietal pleura was significantly shorter in mycoses as compared to other lesions (5.1 ± 9.3 vs. 14.4 ± 16.6 mm), and the width of the contact area between parietal pleura and the lesion was larger (33.9 ± 61.2 vs. 19.5 ± 35.6 mm; *p* < 0.0001 each). The overall pneumothorax rate was 16%, but in only 2% of all cases the placement of a chest tube was indicated. In 77% of the cases a visible bleeding occurred after the biopsy, and the size of the hematoma averaged between 25.9 ± 34.2 and 14.4 × 16.2 mm; in two cases, the procedure had to be discontinued due to serious bleeding, one self-limiting and the other treated by bronchoscopic treatment. One case of fatal air embolism occurred [14, 15].

Patients with proven fungal infections had a slightly higher number of biopsies taken than patients without mycosis (8.5 ± 3.3 versus 7.7 ± 3.3, *p* = 0.029). Biopsy numbers in patients suffering from IFDs and major complications was 8.5 ± 0.7 and those with minor complications 6.5 ± 2.8 (*p* > 0.05). However, both were lower than in mycosis patients without complications (8.9 ± 3.3, *p* > 0.05). The number of biopsies taken in IFD patients with any complication was 7 ± 2.6 and slightly lower when compared to the remaining population investigated. The differences between the numbers of biopsies taken in patients with minor or major complications were the same when compared with the number in the normal population (*p* > 0.05 each).

## Discussion

CT-guided percutaneous lung biopsies performed in immunocompromised patients over the last 12 years was associated with a tolerable risk and was an effective diagnostic tool. *Aspergillus* as the leading mold in previous years was replaced by non-*Aspergillus* pathogens likewise as a consequence of effective *Aspergillus* targeting drugs used for mold prophylaxis to prevent IFD.

Although pulmonary infections may be diagnosed by the performance of broncho-alveolar lavages (BALs) as well as GM antigen testing, CT-guided lung biopsies increase the diagnostic yield and accuracy [21], allowing to specify the underlying pathogen and to rule out contamination or noninfectious causes [22]. The sensitivity of the culture and microscopy in diagnosing IA are about 50% in high-risk patients with hematological disease [23], GM detection seems to be more sensitive [24], but sensitivity is variable and decreases tremendously in patients receiving anti-mold prophylaxis [25–27]. These facts limit their unrestricted use, especially from a clinical point of view when pathogen specification is highly warranted for further medical interventions.

The detection of fungi in tissue or other sterile specimens provides definitive diagnosis of IFD, independent of host factors or clinical features. Speed is crucial and CFWS allows a differentiation between infections caused by septate molds (*Aspergillus* spp.) or non-septate molds of the order Mucorales (members of the families *Mucoraceae, Cunninghamellaceae, Saksenaeaceae, Mortierellaceae, and Syncephalastraceae*), or others within 30 min, which is crucial for the choice of proper antifungal treatment [8, 28]. Direct microscopy is especially important for the diagnosis of non-septate fungi, because these fungi are poorly recovered by culture [9] and first-line treatment consists of polyene therapy. For IA, the recommended drug of choice is voriconazole, which lacks activity against Mucorales. At the time of diagnosis, 55% of our patients with proven mucormycosis received non-Mucorales-active drugs as primary therapy. At our institution, infections due to *A. terreus* were prevalent within the last few years; however, the timely use of voriconazole and the widespread use of micafungin and/or posaconazole as prophylaxis decreased IA in general including *A. terreus* infections; see Table 4. Nowadays, *Aspergillus* has been more and more replaced by Mucorales or other fungal pathogens. We speculate that this epidemiological situation is due to the extensive application of *Aspergillus*-active drugs mainly as prophylaxis. In 2014, there was no single case of proven IA in patients with underlying hematological malignancies, yet infections due to rare fungal pathogens albeit in a low frequency were detected. These fungal pathogens are challenging, as the best treatment options are not yet known. *Fusarium* spp. and *Scedosporium* spp. may not be distinguishable from *Aspergillus* spp. by hyphal morphology in tissue, but may require different management [28]. The proper identification of the fungal genus is therefore highly warranted. The application of *Aspergillus* PCR, broad-range fungal PCR, and GM detection, or any combination of the above, resulted in an improved, fast, and powerful fungal specification in CFWS-positive samples. 100% specificity and sensitivity was achieved by using these tests in combination (data not shown). The culture technique was less helpful, as 68% of specimens remained negative.

In our study, 127 immunocompromised patients were highly suspicious for IFD due to pathological CT findings; CT-guided biopsies confirmed the diagnosis in 81%. CT scan sensitivity, specificity, NPV, and PPV were 100, 44, 100, and 80%, respectively. So far, CT findings related to fungal infections have been reported in association with other pathogens such as *Herpes simplex*, *Cytomegalovirus*, and *Mycobacterium tuberculosis* [29, 30]. In our study, CT-guided biopsy revealed negative results for fungi in 30 (19%) patients. In 12 cases, other clinical diagnoses, such as lung carcinoma, leukemic infiltrates, or tuberculosis, were confirmed by pathological examinations. In three cases, however, fungal infection was considered despite negative CFWS. The clinical course and response to antifungal therapy confirmed mycosis being eventually present. Overall, the rate of false-negative results might be low upon examination of biopsy specimens. McCabe reported two cases (13%) which were undetected by an open lung biopsy; similar numbers were found by Cheson et al. [31]. False-negative results may be explained by sampling errors or biopsies obtained in marginal zones of tissue reorganization and necrosis [22]. Therefore, multiple specimens should be taken from different representative regions of the lesions in cases of suspicion of mycosis. A contrast medium-enhancing region should be considered for biopsy, as well as adjacent transition zones to normal parenchyma and to the necrotic zone.

In a retrospective study reviewing the clinical outcome of 17 patients, Nosari et al. [32] showed CT-guided lung biopsies not to be associated with major side effects. Pneumothorax, pulmonary hemorrhage, and fungal dissemination through surgical procedures have previously been reported; however, it is concluded that this radiological intervention is safe [22, 32–34]. Complications may be associated with the size of the lesions and their localization [21]. Unimpaired hemostasis is of utmost importance. In our series, only two bleeding complications with formal consequences occurred. In the first case, the bleeding was self-limiting after a few minutes, while in the second case bronchoscopic intervention was necessary. The risk for severe hemoptyses (8% in our series) may be reduced by using the ipsilateral dependent position. Moreover, we hypothesize that fatal cases of air embolisms have been prevented by using this position [19]. The risk of complications is probably enhanced in patients with severe coagulopathy or with profound thrombocytopenia and in patients on mechanical ventilation. The higher number of biopsies taken in patients with proven fungal infections may be explained by a greater suspicion of IFD being present of the performing radiologist. The more the number of human specimens available, the higher is the diagnostic output. The cause for the slightly lower number of biopsies taken in patients suffering from proven fungal infections with any complication is probably because biopsy procedures are presumably abandoned if difficulties occur.

So far, only an accurate diagnosis permits a targeted therapy, an adequate secondary prophylaxis if needed, and facilitates the decision whether a surgical treatment is indicated or not. CFWS allowed a fast diagnosis and the differentiation between septate and unseptate hyphae, whereas the clinical and CT findings for pulmonary aspergillosis and mucormycosis are similar, respectively [35]. The differences in the frequency of opacities, cavities, halo sign, and air crescent are insignificant between these infections. So far, Chamilos et al. [36] found that the presence of multiple nodules and pleura effusion at the initial CT scan was an independent predictor of pulmonary mucormycoses. Due to the limited number of such cases, we were not able to detect differences between these entities.

Our study has several limitations, as we conducted a single-center study, and the nature of data collection was retrospective. In addition, over time molecular-based techniques changed depending on the samples obtained and studies performed. *Aspergillus* PCR was applied in specimens displaying *Aspergillus*-like hyphae, and the panfungal PCR in cases of non-*Aspergillus*-like hyphae being present. There was no head to head comparison of both methods in the presence of positive fungal elements, and the usage of CFWS has been stable over the years.

In view of our experience, we propose CT-guided percutaneous biopsies in patients suspected of IFD not responding to standard therapy. The application of CFWS was superior in detecting fungal elements and in distinguishing between septate and non-septate hyphae; the combination of GM, *Aspergillus* PCR, and broad fungal PCR resulted in fast and reliable fungus identification. The complication rates were acceptable and comparable to those of previous studies and may outweigh by far the benefits of an accurate diagnosis and a guided specific therapy.
